# Biomechanical influence of TKA designs with varying radii on bilateral TKA patients during sit-to-stand

**DOI:** 10.1186/1476-5918-7-12

**Published:** 2008-08-13

**Authors:** He Wang, Kathy J Simpson, Samatchai Chamnongkich, Tracy Kinsey, Ormonde M Mahoney

**Affiliations:** 1 School of Physical Education, Sport, and Exercise Science, Ball State University, Muncie, IN, USA; 2 Department of Kinesiology, University of Georgia, Athens, GA, USA; 3 Department of Physical Therapy, Chiang Mai University, Chiang Mai, Thailand; 4 Athens Orthopedic Clinic, PA, Athens, GA, USA

## Abstract

**Background:**

Compared to the design of a traditional multi-radius (MR) total knee arthroplasty (TKA), the single-radius (SR) implant investigated has a fixed flexion/extension center of rotation. The biomechanical effectiveness of an SR for functional daily activities, i.e., sit-to-stand, is not well understood. The purpose of the study was to compare the biomechanics underlying functional performance of the sit-to-stand (STS) movement between the limbs containing an MR and an SR TKA of bilateral TKA participants.

**Methods:**

Sagittal plane kinematics and kinetics, and EMG data for selected knee flexor and extensor muscles were analyzed for eight bilateral TKA patients, each with an SR and an MR TKA implant.

**Results:**

Compared to the MR limb, the SR limb demonstrated greater peak antero-posterior (AP) ground reaction force, higher AP ground reaction impulse, less vastus lateralis and semitendinosus EMG during the forward-thrust phase of the STS movement. No significant difference of knee extensor moment was found between the two knees.

**Conclusion:**

Some GRF and EMG differences were evident between the MR and SR limbs during STS movement. Compensatory adaptations may be used to perform the STS.

## Background

During the past decade, the understanding of knee joint kinematics has improved, and the designs of total knee arthroplasty (TKA) systems have progressed tremendously. It was believed that multiple instantaneous centers of knee flexion/extension (KF/KE) rotation existed in normal knee joints [1,2]. However, some investigators [e.g., [3-5]] determined that there was only one KF/KE axis fixed within the femur. Subsequently, some TKA systems were designed with a single-radius (SR) of rotation compared to the traditional TKA designs of multiple radii (MR) of rotation.

One primary biomechanical difference between the SR and the MR designs investigated in this study that could lead to functional performance differences is that the KF/KE axis of the SR compared to those of the MR designs is more posterior [6,7]. Therefore, the knee with an SR design should have a longer quadriceps moment arm than an MR knee. A cadaveric study conducted by D'Lima et al. [3] showed that less quadriceps muscle force was required to produce a 40 Nm knee extension moment with an SR TKA compared to an MR TKA during a dynamic condition simulating stair climbing. It was also found that the unilateral SR TKA patients used less TKA quadriceps activation than that of the MR TKA patients during the stand-to-sit movement [8]. Furthermore, patients with a unilateral SR TKA limb have demonstrated faster movement times, less trunk flexion, and less TKA quadriceps activity than those with a unilateral MR limb during sit-to-stand [9].

In addition, the SR design is surmised to have more varus-valgus stability than the MR design [9]. Due to differing radii of curvature, an abrupt shift from a longer to a shorter radius within an MR design during knee flexion may cause temporary varus-valgus knee instability (OMM, personal observation). For the MR TKAs used in this study, the shift from the first KF/KE axis to the second axis usually occurs between 30° and 45° of clinical knee flexion (0° = full knee extension). Clinically, this axis shift appears to correspond approximately to the knee position where mid-flexion instability occurs to some MR patients during movements of raising or lowering the body. Also, increased antagonistic hamstring activation of the MR compared to SR limb during the stand-to-sit might be a result of the possible knee mid-flexion instability [8].

Although there are kinematic and EMG evidences to show that the unilateral TKA patients with an SR limb can perform the sit-to-stand (STS) activity in less time with less muscular activation than those with an MR limb [9,10], there is a lack of kinetic evidence to demonstrate how the mechanical differences between the SR and MR designs influence the performance of the STS. Therefore, the *primary purpose of this study *is to determine if kinetic differences may underlie the mechanical differences between the SR and MR limbs during the STS movement.

Osteoarthritis is a primary reason for a TKA, and it is common that osteoarthritis influences both legs. Often then, TKA patients eventually have bilateral TKAs. As it is anticipated that the two TKA systems are mechanically different, it is important to know if two mechanically different TKAs influence bilateral TKA patients' performance during daily activities.

The STS is considered to be one of the most mechanical demanding of common daily movements [11,12]. As such, it is often used by orthopedic surgeons to assess function after TKA surgeries because it requires more knee extensor torque than other daily activities, such as level walking and stair climbing [11,12]. Therefore, the *second purpose of this study *was to investigate whether bilateral TKA patients with the mechanically different SR and MR TKAs display compensatory motion during the STS movement.

## Methods

Ten individuals from a group of well-functioning patients who received bilateral TKA surgeries from the same surgeon with a minimum post-operative time of 24 mo. participated in the study. Participants were medically cleared and prescreened for health, injury or balance problems that could affect their performance. Two participants were later eliminated from the study due to uncovered medical history that was not compliant with the screening criteria. This study was approved by our institutional review board. Each participant gave written informed consent before testing. The means and standard deviations of the participants' mass, height, and age were 85.7 ± 9.7 kg, 1.66 ± 0.07 m, and 72.4 ± 9.2 yr, respectively. Each participant had one limb with an SR TKA (Scorpio™ PS, Stryker Orthopaedics, Inc.) and the other contained an MR TKA (S-7000™ PS, Stryker Orthopaedics, Inc. or P.F.C.™ PS, Johnson & Johnson, Inc. for three and five participants, respectively). Table 1 shows the means and standard deviations of the post-operative times, clinical and functional knee society scores, and isometric strength for the SR and MR limbs, respectively. The design similarities and differences between the two MR TKAs are as follows: First, all TKAs were posterior cruciate sacrificing (PS). Second, there are two and three radii in the femoral components of the S-7000™ and P.F.C™ TKAs, respectively. Third, for a medium size implant, the transition from the first long radius (33 mm and 35 mm for S-7000™ PS and P.F.C™ PS, respectively) to the second short radius (19 mm and 21 mm for S-7000™ PS and P.F.C™ PS, respectively) occurs during 30° to 45° of clinical knee flexion. Fourth, the conformity of the tibia insert to the femoral component in the medio-lateral and antero-posterior directions are similar for both MR knees.

**Table 1:** Means (SD) of post-operative time, clinical and functional knee society scores, and isometric peak torques (ISO_PT) of quadriceps and hamstrings of the SR and MR limbs.

	SR Limb	MR Limb
Post-operative time (mo.)	34(8)	86(31)
Clinical Score	93.9(6.9)	94.0(6.9)
Functional Score	88.7(18.0)	88.7(18.0)
ISO_PT of quadriceps (Nm)	150.7 (76.0)	124.3 (23.4)
ISO_PT of hamstrings (Nm)	80.1 (42.3)	98.2 (91.5)

The instrumentation included an electromyography (EMG) system (MYOPAC™: 1080 Hz, CMRR = 110 dB), three high-speed video cameras (Pulnix™: genlocked, 120 Hz, shutter speed = 1/1000 s), one force platform (AMTI™: 1080 Hz), and an isokinetic dynamometer (Kin-Com III™: 500 Hz). The equipment were synchronized through an event video control unit (EVCU) (Peak Performance Technologies, Inc). The EMG system was used to collect differential surface EMG signals of the vastus medialis and lateralis (VM and VL), rectus and biceps femoris (RF and BF), and semitendinosus (ST) while the participant performed the STS movement and maximum effort isometric strength testing. During the STS testing, the cameras were used to track the motions of reflective markers placed on the body. For a given side of the body, markers were placed at the fifth metatarsal head, lateral heel, lateral malleolus, anterior and posterior distal one-third of the tibia, tibial tuberosity, lateral and medial femoral epicondyles, lateral mid thigh, greater trochanter, iliac spine, and the acromion process. The force platform was placed under one foot at a time and used to collect the ground reaction forces (GRF). The isokinetic dynamometer was used for conducting separate isometric strength testing of the knee extensor and flexor muscles in order to obtain the isometric maximum voluntary actions. Participants came to two accommodation sessions to practice the isometric strength testing, as it has been shown that is requires this number of sessions to obtain the true maximum strength using this instrument [13]. At the third session, the participant performed the STS task before the isometric test. For the STS task, the participant sat on a wooden box, with the box height adjusted so both thighs were parallel to the floor at approximately 90° of knee flexion. The goal of the task was then to stand up as rapidly as possible and then remain standing (5 s). The right side of the body was tested first. A total of four trials were administered for each limb. Next, using maximum voluntary effort (MVE), the isometric strength test was administered for knee extensors (60°) and flexors (30°) using standard procedures proposed by Perrin [14].

For data reduction, the raw EMG signals were bandwidth filtered (10 Hz – 1000 Hz). The EMG data collected during the MVE trial with the highest isometric torque measured were used to scale the STS RMS data. The EMG values from the STS trials were rectified and scaled to MVE EMG, and then the root-mean square (RMS) data were produced (T = .026 s). For each of the two STS phases, forward-thrust phase (from beginning of the STS to the maximum trunk flexion angle), and extension phase (from the maximum trunk flexion angle to the end of the STS), the mean of the scaled RMS displayed during that phase was calculated.

Reflective markers were automatically digitized through Peak Motus™ program, and the spatial locations were visually checked for all video fields prior to filtering the raw coordinates. Three-dimensional (3D) coordinates were constructed by using modified Direct Linear Transformation (DLT) method through Peak Motus™ software. Raw three-dimensional (3D) coordinate data were smoothed (quintic spline [15]), and sagittal plane kinematics and kinetics were generated. A vector approach was used to calculate angular kinematics, which included ankle, knee, hip, and trunk angles and angular velocities. For the angular kinetics, an inverse dynamics approach was used to calculate lower extremity flexor/extensor joint moments [16].

Primary kinetic variables included peak values of antero-posterior GRF (AP-GRF) and vertical GRF (VGRF), knee joint moment, and GRF impulses in the AP and vertical directions during the forward thrust and extension phases of the STS. Primary EMG variables included the average, scaled RMS EMG of VM, VL, RF, BF, and ST for the two STS phases. The following kinematic variables for the ankle, knee and hip joints were secondary variables, that is, only used for descriptive purposes: angular displacements and maximum extension velocities. In addition, isometric strength data of knee flexor and extensor were used for descriptive purpose.

For each variable, average value of the four STS trials were calculated and used for statistical analysis. Shapiro-wilk test was used to check normality of the data. The SR and MR limbs were compared for the primary variables using paired Student's t-tests. (*p *< 0.05). As the small sample size (n = 8) used in this study limited the statistical power, we considered that there was a tendency for inter-limb differences if the *p *value was greater than 0.05 but less than 0.10 and did not correct for family-wise Type I error.

## Results

**Figure 1:**
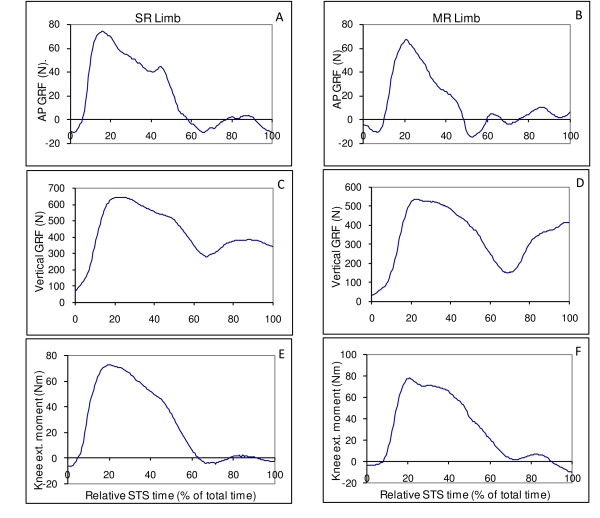
**Sample graphs from one participant**. Graphs A, C, and E are SR limb's antero-posterior GRF, vertical GRF, and knee extension moment, respectively. Graphs B, D, and F are MR limb's antero-posterior GRF, vertical GRF, and knee extension moment, respectively.

Data collected in this study were normally distributed. For the GRF, significant differences were found only for AP-GRF variables between the limbs (Table 2). The SR limb had 10 N more peak AP GRF than the MR limb (*p *= 0.007) during the forward-thrust phase (Table 2). The SR limb also had greater AP ground reaction impulse (13.9 ± 3.6 N·s) during forward-thrust phase than that of the MR limb (10.4 ± 3.5 N·s) (*p *= 0.006). No significant differences were displayed for the peak knee extensor moment between the SR and MR limbs (Table 2). Qualitatively, both TKA limbs displayed a similar pattern of knee extensor moment (Figure 1).

**Table 2:** Means (SD) and *p *values of peak antero-posterior (AP) and vertical ground reaction forces (GRF) (N), knee joint moment (Nm/kg), AP and vertical ground reaction impulses (Ns) during forward-thrust phase and extension phase of the STS between the SR and MR limbs.

	SR	MR	*p *values
Peak AP GRF	60.5 (8.5)	50.3 (10)	0.0066
Peak vertical GRF	479.3 (74.5)	486.3 (51.3)	0.38
Peak knee extensor joint moment	0.75 (0.19)	0.79 (0.15)	0.29
AP impulse during forward-thrust phase	13.9 (3.6)	10.4 (3.5)	0.0059
Vertical impulse during forward-thrust phase	127.9 (26.1)	124.2 (25.0)	0.28
AP impulse during extension phase	8.0 (2.4)	7.1 (3.6)	0.31
Vertical impulse during extension phase	286.9 (80.9)	302.6 (136.4)	0.36

**Figure 2:**
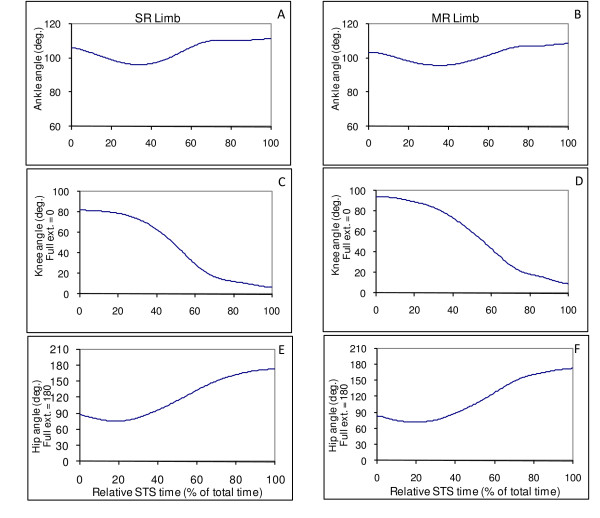
**Sample graphs from one participant**. Graphs A, C, and E are SR limb's ankle, knee, and hip angles, respectively. Graphs B, D, and F are MR limb's ankle, knee, and hip angles, respectively.

For the angular kinematics, Figure 2 demonstrates similar patterns of ankle, knee, and hip joint angular displacements between the two TKA limbs during the STS for a representative participant. The descriptive data from Table 3 also qualitatively exhibits a tendency of similarity between the two TKA limbs for peak angular velocities of ankle, knee, and hip joints during the STS.

**Table 3:** Means (SD) of peak angular velocities (vel.) (deg/s) of ankle, knee, and hip joints between the SR and MR limbs during the STS.

Peak angular velocity	SR	MR
Ankle plantar-flexion	29(12)	32(10)
Ankle dorsi-flexion	42(14)	42(15)
Knee extension	151(36)	153(25)
Hip extension	73(7)	79(12)
Hip flexion	155(28)	164(16)

For the scaled, RMS EMG during the STS (Table 4), the VL of MR was significantly greater (*p *= 0.03) or tended to be greater (*p *= 0.078) than that of the SR limb during the forward-thrust and extension phases, respectively. The ST activity of the MR limb tended to be greater than that of the SR limb during forward-thrust (*p *= 0.07). In addition, 6 out of 8 participants displayed greater ST activity of the MR limb than the SR limb. No other muscles displayed significant EMG differences between TKA limbs.

**Table 4:** Means (SD) and *P *values of average RMS EMG of VM, VL, RF, BF, and ST during forward-thrust phase and extension phase of the STS between the SR and MR limbs.

	SR	MR	*p *values
VM in forward-thrust	1.412 (0.833)	1.561 (0.603)	0.28
VL in forward-thrust	1.185 (0.508)	1.354 (0.631)	0.03
RF in forward-thrust	1.061 (0.450)	1.314 (0.649)	0.17
BF in forward-thrust	0.398 (0.223)	0.451 (0.474)	0.405
ST in forward-thrust	0.277 (0.200)	0.331 (0.247)	0.07
VM in extension	0.881 (0.459)	0.971 (0.437)	0.228
VL in extension	0.813 (0.389)	0.904 (0.408)	0.078
RF in extension	0.496 (0.186)	0.549 (0.218)	0.317
BF in extension	0.435 (0.202)	0.440 (0.404)	0.485
ST in extension	0.258 (0.118)	0.325 (0.241)	0.176

## Discussion

Our main research question was "Do MR compared to SR limbs display kinetic differences to accomplish the STS movement or does the MR limb require greater levels of knee extensor activation to create the necessary knee joint moments?" As the moment arm length for the quadriceps force acting on the tibia via the patella tendon has been shown to be longer for the SR than the MR TKA designs used in this study [3,7], we had expected that, in comparison to the MR limb, the SR limb would either produce: a) more knee extensor torque with similar quadriceps activation or b) generate equal knee extensor torque but require less quadriceps activation.

There is limited support for the second predicted outcome. There was no significant inter-limb difference for peak knee extensor moment. Although it cannot be said that the limbs displayed similar values, the inter-limb difference for the peak knee extensor moment was only 0.044 Nm/kg. Furthermore, the SR limb used less VL activation to generate the joint moments required to raise the body during the initial phase of the STS than the MR limb. Why the VL was the only knee extensor muscle to display significant inter-limb differences is not known, but it is similar with prior outcomes [9]. Less VL activation to extend the knees to raise the body was displayed by unilateral TKA individuals with an SR compared to an MR TKA [9]. In addition, it is interesting to note that during the forward-thrust phase of the STS, the SR limb produced greater peak anterior GRF, hence also greater anterior GRF impulse than the MR limb. During this time, the trunk segment is flexed around the hip joints so that the upper body weight can be shifted anteriorly from the chair seat to a line of action through the feet. Thus, the SR limb contributed a greater proportion of anterior impulse to generate anterior body momentum to facilitate trunk rotation and therefore help the TKA patient to stand up. It has been reported that to reduce the difficulty of the STS movement, TKA patients compared to matched control participants use more trunk flexion during the forward thrust phase [12] to compensate for extensor muscle weaknesses of the TKA limbs. Consistent with those findings, Wang, et al [9] observed that for unilateral participants, MR individuals used greater trunk flexion and tended to have greater trunk flexion velocity than SR individuals.

As we believe that multiple radii may differentially influence the tension of passive tissue surrounding the knee joint [8], we had surmised that the MR compared to the SR limb would exhibit increased hamstrings activation to increase the stability of the knee joint. Only tendency of increased antagonist activation by the ST of the MR limb was found (*p *= 0.07) during the forward-thrust phase. However, six out of eight participants showed greater ST activation for their MR limbs during the STS. This suggests that a majority of the individuals increase ST activation to stabilize the knee during the STS.

The second purpose of this study was to observe whether patients with two mechanically different TKA systems perform the STS showed kinematic compensatory adaptation. After qualitatively reviewing the kinematic data and graphs, we found similar patterns of ankle, knee, and hip angular displacements and angular velocities between the two limbs. Although some significant between-limb differences for AP GRF and EMG patterns were found between the TKA limbs during the STS, we suggest that the movement likely was performed in a kinematically symmetric fashion. We believe that, in general, performers try to maintain inter-limb kinematic symmetry during bilateral movements to improve stability and effectiveness of the movement.

The interpretations of our outcomes are constrained by several potential limitations. First, a relatively small sample size (8 participants) reduced the statistical power. Second, the post-operative time of the MR limb was significantly longer than the SR limb (*P *= 0.003), but we believe the effects are minimal. Post-operative time for both TKA limbs, on average, was more than two years. Hence, enough time had passed for the strength of both TKA knees to have recovered sufficiently [17,18]. Also, both Knee Society scores of both limbs were excellent and did not vary by more than one point. The third limitation is that two different types of MR TKA (S-7000™ PS, Stryker Orthopaedics, Inc. and P.F.C.™ PS, Johnson & Johnson, Inc.) were used in the study. Different MR designs may influence the MR limbs differently. However, important elements of these designs provided in the methods are comparable. It was surmised that the design differences between the SR and MR knees were of greater magnitude and behavioral significance than differences between the two MR TKAs. The fourth limitation was that the limbs were tested during different trials. This reduced our ability to investigate interlimb compensatory strategies. We believe, however, that the trials obtained for each limb were typical STS trials and not biased toward one limb or the other, as the averages of the rise times of the MR and SR trials were within 0.09 s of one another.

## Conclusion

The SR compared to MR limbs displayed greater AP GRF and AP ground reaction impulse with less VL activation during the sit-to-stand. Future work will allow us to determine if the increased VL displayed by MR limbs was to compensate for a reduced quadriceps moment arm, and the increased ST activation associated with the MR limbs likely increased knee stability.

## Competing interests

The authors declare that they have no competing interests.

## Authors' contributions

HW conceived the study, participated in study design, data collection, calculation, and statistical analysis, drafted and revised manuscript. KS conceived the study, participated in study design, helped draft and revise the manuscript, gave final approval of the version to be published. SC participated in data acquisition and helped draft the manuscript. TK participated in study design and coordination, recruited and assessed potential participants and helped draft the manuscript. OM conceived the study, participated in study design, recruited and assessed potential participants, and helped draft the manuscript. All authors read and approved the final manuscript.
